# The Relationship between Perirenal Fat Thickness and Reduced Glomerular Filtration Rate in Patients with Type 2 Diabetes

**DOI:** 10.1155/2020/6076145

**Published:** 2020-06-28

**Authors:** Yuan Fang, Yuechao Xu, Yuxian Yang, Chang Liu, Dong Zhao, Jing Ke

**Affiliations:** ^1^Center for Endocrine Metabolism and Immune Diseases, Beijing Luhe Hospital, Capital Medical University, Beijing 101149, China; ^2^Beijing Key Laboratory of Diabetes Research and Care, Beijing 101149, China

## Abstract

**Background:**

Obesity has been considered as an important factor in the development and progression of chronic kidney diseases (CKD). Perirenal fat, which is surrounding the kidneys, has been reported to be unique in anatomy and biological functions. This study is aimed at assessing the relationship between perirenal fat thickness (PrFT) and estimated glomerular filtration rate (eGFR) in patients with type 2 diabetes (T2DM).

**Methods:**

A total of 171 patients with T2DM were recruited in the study. The basic and clinical characteristics including sex, age, diabetes duration, body mass index (BMI), waist circumference (WC), visceral fat area (VFA), glycated hemoglobin (HbA1c), serum uric acid (UA), total cholesterol (TC), low-density lipoprotein cholesterol (LDL-c), and high-density lipoprotein cholesterol (HDL-c) were collected. PrFT was measured via ultrasound. eGFR was calculated using the Modification of Diet in Renal Disease (MDRD) formula.

**Results:**

Patients were divided into three groups according to PrFT, and we found patients with higher PrFT had lower eGFR. PrFT was significantly correlated with eGFR in all patients (*r* = −0.181, *P* < 0.05). Subgroup analysis by sex showed that PrFT still significantly and negatively related to eGFR in men (*r* = −0.264, *P* < 0.05), but not in women (*r* = −0.199, *P* = 0.062). The association also existed in multivariate analysis after correction for the confounding factors (*β* = −0.203, *P* = 0.017).

**Conclusions:**

This study confirmed a negative independent relationship between PrFT and eGFR in patients with T2DM, especially in men, suggesting a possible role of perirenal fat in kidney dysfunction in T2DM patients.

## 1. Introduction

Obesity has been recognized as a major public health problem and caused economic burden [1]. Obesity is associated with the development and progression of chronic kidney disease (CKD), and multiple mechanisms including chronic inflammation, increased oxidative stress, and hyperinsulinemia have been proposed to initiate and maintain kidney injury in obese patients [2, 3]. Diabetic kidney disease is one important complication of diabetes. Except for hyperglycemia, many factors including obesity have been reported to be risk factors for diabetic kidney disease [4, 5].

Body mass index (BMI) and waist circumference (WC), widely used as obesity indexes, have been proved to aggravate the development of CKD in different subsets of patients [6–8]. However, BMI could not distinguish the muscle from the fat content [9, 10], and subjects with similar BMI level may have different metabolic and cardiovascular risk (CVD) [11]. In addition, BMI and WC lack detailed information about the distribution of body fat. Body fat is now usually divided into subcutaneous fat and visceral fat according to the anatomical and physiological functions of fat deposition [12]. Visceral fat has been thought to be associated with the progression of CKD [13–16].

Recently, new anthropometric and imaging methods have been used to assess visceral fat in clinical practice and research. Perirenal fat is a fat pad surrounding the kidneys, located between the renal fibrous membrane and the renal fascia in the retroperitoneal space [17]. While paranephric fat is anatomically adjacent to perirenal fat, the renal fascia separates perirenal fat from paranephric fat [17], whereas sinus fat is perivascular fat, confined to the renal sinus and the renal fibrous membrane [18]. Anatomy studies have confirmed that perirenal fat has a complete system of blood supply, lymph fluid drainage, and innervation compared to classically connective tissues [19–21]. Histologically, paranephric fat is a typical white adipose tissue depot, while perirenal fat mainly consists of dormant brown adipose tissue [22]. Besides, perirenal fat is more active in energy metabolism and adipokine secretion compared with typical visceral fat [17, 23]. Renal sinus fat has been proved to increase in patients with prediabetes and diabetes, and negatively associated with estimated glomerular filtration rate (eGFR) in patients with type 2 diabetes mellitus (T2DM) [24, 25]. Additionally, it has been proved that renal sinus fat was associated with an increased risk of CKD in general subjects through inflammatory signaling [26–28]. Some researchers have shown that massive perirenal fat thickness (PrFT) was an early predictor of atherosclerosis [29]. De Pergola et al. have found a positive association between paraperirenal fat thickness and mean 24 h diastolic blood pressure levels in overweight and obese subjects [30]. Another study suggested the close relationship between perirenal fat and microalbuminuria and reduced glomerular filtration rate (GFR) in obese rats and obese swine [31, 32].

Based on the above background, our study is aimed at assessing the relationship between PrFT and eGFR in patients with T2DM.

## 2. Materials and Method

### 2.1. Patients

A total of 171 inpatients visiting the department of endocrinology of Beijing Luhe Hospital, Capital Medical University, were recruited from September 2019 to October 2019. Patients with renal replacement therapy (renal transplant or dialysis patients), renal morphological abnormalities (difference in kidney length between the two kidneys > 1.5 cm, solitary kidney or multiple kidneys, congenital kidney abnormalities, polycystic kidney, and hydronephrosis), liver cirrhosis, chronic obstructive lung disease, previous diagnosis of a tumor, and present pregnancy were excluded. The present study was carried out in accordance with the Declaration of Helsinki, and the study protocol was approved by the ethical committee of Beijing Luhe Hospital.

### 2.2. Anthropometric Evaluation

All subjects participating in our study underwent a physical examination including measurements of height, weight, and WC. BMI was calculated as weight divided by square height (kg/m^2^). Serum creatinine, total cholesterol (TC), high-density lipoprotein cholesterol (HDL-c) and low-density lipoprotein cholesterol (LDL-c), triglycerides (TG), uric acid (UA), fast plasma glucose (FPG), and glycated hemoglobin (HbA1c) were examined. eGFR is calculated using the MDRD formula: [eGFR (ml/min/1.73 m^2^) = 175 × (Scr/88.4)^−1.154^ × (Age)^−0.203^ × (0.742 if female)]. Urinary microalbumin and creatinine concentrations were determined on the morning of the clinical examination using an early-morning first sterile urine sample with the electrochemical luminescence methods (Roche Diagnostics GmbH, Germany). The urinary microalbumin to creatinine ratio (UACR) was then calculated by a machine (UACR = urinary microalbumin/urinary creatinine × 8841). Visceral fat area (VFA) was estimated through DUALSCAN HDS-2000, an abdominal dual BIA machine (OMRON Healthcare Co., Kyoto, Japan).

### 2.3. Measurement of Perirenal Fat Thickness

Ultrasound examinations were performed by a single skilled operator who did not know the patient's clinical data, using a duplex Doppler apparatus (Model Preirus, HITACHI). PrFT and paranephric fat thickness (PnFT) were measured while the patient was in a supine position. The probe was held vertical to the skin on the transverse aspects of the abdomen and slowly moved laterally until the optimal position was found. Longitudinal scanning was performed with the kidney surface almost parallel to the skin. The pressure on the probe should be as small as possible to prevent the fat layer from being compressed. PrFT and PnFT were then determined from the inner side of the abdominal musculature to the surface of the kidney. The average of bilateral ultrasound measurements was calculated as the PrFT and PnFT.

### 2.4. Statistical Analysis

The population was divided into three groups based on quartiles of PrFT (PrFT1 < 0.6 cm, PrFT2 0.6 − 1.33 cm, PrFT3 ≥ 1.33 cm). Continuous variables were expressed as mean ± SD. Skewed variables were given as a median and interquartile range. Differences between groups were evaluated using analysis of variance (ANOVA). A Chi-square test was used to examine the difference between groups for categorical variables. A nonparametric test was used for Skewed variables.

The univariate relationships were tested by simple linear regression analyses. Then, subgroup divided by sex was analyzed. Multiple linear regression analysis models were carried out considering eGFR as an outcome variable and correct potential confounding factors: age, diabetes duration, FPG, TC, LDL-c, HbA1c, and PrFT. The statistical analyses were performed using the IBM SPSS Statistics software package (version 25 for Windows, Chicago, IL) and statistical package R (version 3.5.2, available from http://www.r-project.org).

## 3. Results

### 3.1. Clinical Characteristics of the Subjects

Table 1 shows the characteristics of the overall study subjects as well as divided into three groups according to quartiles of PrFT. The mean value of PrFT in the 171 patients with T2DM was 0.97 ± 0.50 cm. There were significant differences in BMI, WC, VFA, UA, TG, and HDL-c among three groups. Subjects with higher PrFT had higher UA and TG and lower HDL-c levels compared to those with lower PrFT (*P* < 0.01). And subjects in the higher PrFT group had higher BMI, WC, VFA, and PnFT compared to those in the lower PrFT group (*P* < 0.01). Moreover, subjects in the uppermost PrFT group had the lowest eGFR than the other two groups (*P* < 0.05). But no significant differences were found in UACR, FPG, or HbA1c among the three groups.

### 3.2. Correlations of Anthropometric and Obesity Parameters

The univariate correlations between obesity parameters including PrFT, PnFT, BMI, WC, and VFA with anthropometric parameters in the entire study population are shown in Table 2. PrFT was strongly and positively associated with other obesity parameters including PnFT, BMI, WC, and VFA. PrFT was significantly and negatively correlated with eGFR in the overall subjects (*r* = −0.181, *P* < 0.05), whereas no correlation was found between eGFR and PnFT (*r* = −0.019, *P* > 0.05), WC (*r* = 0.028, *P* > 0.05), and VFA (*r* = 0.033, *P* > 0.05). Reversely, BMI was significantly and positively associated with eGFR (*r* = 0.166, *P* < 0.05). PrFT was also significantly and positively associated with metabolic parameters, such as UA (*r* = 0.269, *P* < 0.01) and TG (*r* = 0.237, *P* < 0.01), and negatively with HDL-c (*r* = −0.324, *P* < 0.01).

The differences in parameters between men and women are shown in Supplementary Table 1. Men were younger and had shorter diabetes duration. Higher levels of WC, VFA, PrFT, UA, and lower HDL-c were seen in men than in women.

**Figure 1:**
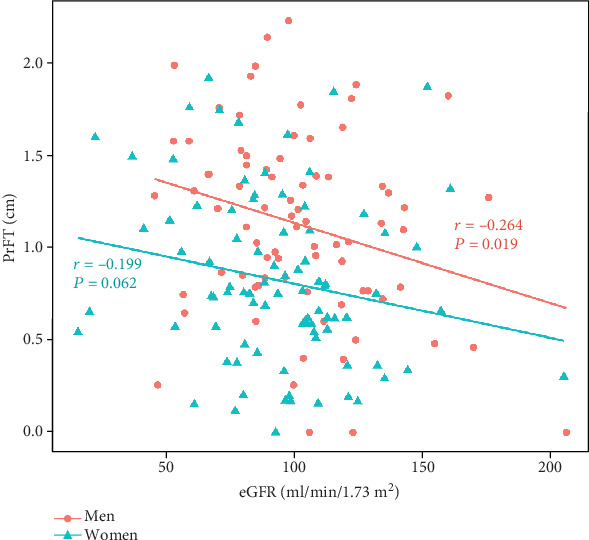
Correlations between perirenal fat thickness (PrFT) and estimated glomerular filtration rate (eGFR) in the population divided by sex.

Then, we analyzed the correlations of eGFR and parameters in subgroup divided by sex (Table 3). The correlations between eGFR and PrFT remained significant in men (*r* = −0.264, *P* = 0.019), whereas became insignificant in women (*r* = −0.199, *P* = 0.062) (Figure 1). eGFR was also significantly positively related to FPG (*r* = 0.249, *P* < 0.05), TC (*r* = 0.287, *P* < 0.05), LDL-c (*r* = 0.273, *P* < 0.05), and HbA1c (*r* = 0.376, *P* < 0.01) and negatively to age (*r* = −0.658, *P* < 0.01) and diabetes duration (*r* = −0.441, *P* < 0.01) in men. eGFR significantly positively correlated with HbA1c (*r* = 0.300, *P* < 0.01) and negatively with age (*r* = −0.546, *P* < 0.01), diabetes duration (*r* = −0.262, *P* < 0.05), and UA (*r* = −0.305, *P* < 0.01) in women.

### 3.3. Multivariate Analysis after Correction for the Confounding Factors

We further established a multivariate model considering eGFR as an outcome variable and independent variables including PrFT, age, diabetes duration, FPG, TC, LDL-c, and HbA1c in men. The results showed that only PrFT, age, and HbA1c were independently correlated with eGFR in men (Table 4). The overall determination coefficient (*R*^2^) was 0.513. Indeed, in the final model, PrFT ranked second among the contributing variables of eGFR (*β* = −0.203, *P* < 0.05), while the strongest independent variable was age (*β* = −0.593, *P* < 0.01).

## 4. Discussion

The most important finding of our study is that PrFT is negatively correlated with eGFR in T2DM patients, and this correlation remains significant after adjusting many confounding factors in men. Several studies have explored the relationship between paraperirenal fat and eGFR in patients with hypertension or obesity, while the results were inconsistent. Paraperirenal fat thickness has been found significantly and negatively correlated with eGFR in patients with hypertension [33]. However, no significant relationship was found between paraperirenal fat thickness and eGFR in obese patients without diabetes or hypertension [34]. Lamacchia and colleagues have demonstrated that paraperirenal fat thickness was independently and negatively associated with eGFR in 151 patients with T2DM [35]. Considering the differences in the structure and function of perirenal fat and paranephric fat, our study analyzed PrFT and PnFT separately and finally found that it was PrFT related to eGFR rather than PnFT. This is mainly due to that perirenal fat directly surrounds the kidney and has a complete system of blood supply, lymph fluid drainage, and innervation compared with other fat depots, which contributes to the uniqueness of perirenal fat. Perirenal fat may influence renal function through mechanical and also paracrine effects [17].

Many investigators have explored the association between PrFT and metabolism. PrFT has been confirmed to be related to metabolic risk factors such as UA, HDL-c, and TG [35], which is consistent with our results. Another study has reported that perirenal fat could exacerbate glucose metabolism through adiponectin in diabetic mice [36], while no significant relation was seen in our study. The widely use of hypoglycemia agents in our study may explain the inconsistency. One study by Lamacchia and colleagues suggested that there was no significant relationship between paraperirenal fat thickness and LDL-c in patients with T2DM [35], similar result was found between PrFT and LDL-c in our research as well.

Increasing evidence has suggested that the accumulation of ectopic lipids around organs may eventually cause organ dysfunction through local mechanisms [37]. First, perirenal fat may influence the function and metabolism of the kidney via secreting paracrine substance. One study showed that perirenal fat induced renal artery vascular endothelial dysfunction partly via tumor necrosis factor-*α* in obese swine [32]. Li et al. have also demonstrated that leptin, an adipocytokine released from perirenal fat, had an adverse impact on kidney damage in metabolic syndrome rat [38]. Another study found that perirenal fat was related to the structural and functional changes of glomerular mesangial cells, podocytes, and proximal tubule cells in mice with obesity-related glomerulopathy [39]. Moreover, perirenal fat could impair organ function through increasing interstitial hydrostatic pressure and reducing renal blood flow by simple physical compression on renal vessels and parenchyma [40, 41].

In addition, our results showed the difference in the correlation between PrFT and eGFR in men and women. PrFT was significantly and negatively correlated with eGFR in men, but not in women. Previous research has showed that patients with comparable WC had higher PrFT in men than in women [42]. In our study, women had lower levels of WC, VFA, and PrFT, lower UA, and higher HDL-c than men. Therefore, we speculate that due to relatively less severe obesity and metabolic disorders, no statistically significant correlation was found between PrFT and eGFR in women.

There are some limitations in our study as well. First, our study is cross-sectional designed, it could not establish a causal relationship between PrFT and eGFR. Further studies are needed to verify these hypotheses. Second, we used estimated GFR instead of measured GFR, which is the most exact and direct indicator of renal function. Third, we measured PrFT and PnFT by ultrasonography instead of computed tomography (CT). Ultrasonography and CT are both widely used in clinical studies. Since ultrasonography is more convenient, fast to measurement, and without radiation, it is widely utilized in clinical practice. According to a previous study, the intraoperator coefficient of variation was 4.5% [33]. Last, we estimated VFA by BIA instead of a standard method such as DXA scanning or CT, which is the most accurate and reliable method. However, VFA analysis through BIA correlates well with that through CT [43]and has the advantage of easiness, inexpensiveness, and avoiding exposure to radiation.

## 5. Conclusions

In conclusion, this study showed PrFT was independently and negatively correlated with eGFR, especially in men, suggesting a possible role of PrFT in kidney dysfunction in T2DM patients.

## Figures and Tables

**Table 1:** Characteristics of the overall study population and of the population divided into three groups based on PrFT.

	Total	PrFT1 (<0.6 cm)	PrFT2 (0.6-1.33 cm)	PrFT3 (≥1.33 cm)	*P*
Sex (men/women)	81/90	12/32	42/44	27/14	0.002
Age (year)	58.14 ± 16.35	59.43 ± 17.67	56.93 ± 15.64	59.29 ± 16.55	0.625
Diabetes duration (year)	10 (3­15)	12 (3.25­16.75)	8 (3­13)	12 (3­20)^#^	0.034
BMI (kg/m^2^)	26.61 ± 4.58	23.31 ± 3.97	27.15 ± 3.67^∗∗^	28.85 ± 5.12^∗∗^^#^	<0.001
WC (cm)	96.84 ± 11.50	87.44 ± 8.77	98.55 ± 9.39^∗∗^	103.26 ± 12.19^∗∗^^#^	<0.001
FPG (mmol/l)	8.60 ± 3.80	8.45 ± 3.83	8.93 ± 4.08	8.11 ± 3.17	0.519
UA (*μ*mol/l)	338.29 ± 99.59	307.81 ± 93.94	336.61 ± 99.22	376.29 ± 96.33^∗∗^^#^	0.008
TG (mmol/l)	1.44 (1.05­2.04)	1.15 (0.84­1.77)	1.44 (1.20­2.06)^∗^	1.75 (1.24­2.85)^∗∗^	0.004
TC (mmol/l)	4.32 ± 1.30	4.61 ± 1.30	4.31 ± 1.30	4.02 ± 1.28^∗^	0.133
HDL-c (mmol/l)	1.06 ± 0.27	1.21 ± 0.26	1.02 ± 0.25^∗∗^	0.98 ± 0.27^∗∗^	<0.001
LDL-c (mmol/l)	2.82 ± 1.04	2.97 ± 1.07	2.84 ± 1.03	2.62 ± 1.03	0.306
HbA1c (%)	9.34 ± 2.15	9.82 ± 2.67	9.41 ± 1.97	8.66 ± 1.71^∗^	0.051
VFA (cm^2^)	110.19 ± 41.79	79.18 ± 30.30	115.39 ± 35.38^∗∗^	142.66 ± 43.23^∗∗^^##^	<0.001
PnFT (cm)	1.00 ± 0.44	0.65 ± 0.33	1.05 ± 0.33^∗∗^	1.28 ± 0.48^∗∗^^##^	<0.001
PrFT (cm)	0.97 ± 0.50	0.36 ± 1.92	0.95 ± 0.22^∗∗^	1.64 ± 0.23^∗∗^^##^	<0.001
UACR (mg/g)	9.18 (3.91­41.94)	9.59 (3.80­26.42)	8.58 (3.39­41.23)	9.29 (4.83­67.28)	0.663
eGFR (ml/min/1.73 m^2^)	97.08 ± 31.11	105.15 ± 36.01	97.78 ± 28.70	86.92 ± 28.14^∗∗^	0.026

BMI: body mass index; WC: waist circumference; FPG: fast plasma glucose; UA: uric acid; TG: triglyceride; TC: total cholesterol; HDL-c: high-density lipoprotein-cholesterol; LDL-c: low-density lipoprotein-cholesterol; HbA1c: glycated hemoglobin; VFA: visceral fat area; PnFT: paranephric fat thickness; PrFT: perirenal fat thickness; UACR: urinary albumin creatinine ratio; eGFR: estimated glomerular filtration rate. PrFT1 vs. PrFT2: ^∗^*P* < 0.05, ^∗∗^*P* < 0.01. PrFT1 vs. PrFT3: ^∗^*P* < 0.05, ^∗∗^*P* < 0.01. PrFT2 vs. PrFT3: ^#^*P* < 0.05, ^##^*P* < 0.01.

**Table 2:** Main correlations of anthropometric and visceral fat parameters in the entire study population.

	PrFT (cm)	PnFT (cm)	BMI (kg/m^2^)	WC (cm)	VFA (cm^2^)
	*r*	*r*	*r*	*r*	*r*
PrFT (cm)	1	0.523^∗∗^	0.424^∗∗^	0.457^∗∗^	0.573^∗∗^
PnFT (cm)	0.523^∗∗^	1	0.552^∗∗^	0.502^∗∗^	0.577^∗∗^
BMI (kg/m^2^)	0.424^∗∗^	0.552^∗∗^	1	0.838^∗∗^	0.776^∗∗^
WC (cm)	0.457^∗∗^	0.502^∗∗^	0.838^∗∗^	1	0.797^∗∗^
VFA (cm^2^)	0.573^∗∗^	0.577^∗∗^	0.776^∗∗^	0.797^∗∗^	1
UA (*μ*mol/l)	0.269^∗∗^	0.231^∗∗^	0.263^∗∗^	0.288^∗∗^	0.486^∗∗^
TG (mmol/l)	0.237^∗∗^	0.292^∗∗^	0.317^∗∗^	0.238^∗∗^	0.404^∗∗^
HDL-c (mmol/l)	-0.324^∗∗^	-0.222^∗∗^	-0.180^∗^	-0.217^∗∗^	-0.254^∗^
eGFR (ml/min/1.73 m^2^)	-0.181^∗^	-0.019	0.166^∗^	0.028	0.033
Diabetes duration (year)	-0.005	-0.289^∗∗^	-0.248^∗∗^	-0.105	-0.185

PrFT: perirenal fat thickness; PnFT: paranephric fat thickness; BMI: body mass index; WC: waist circumference; VFA: visceral fat area; UA: uric acid; TG: triglyceride; HDL-c: high-density lipoprotein-cholesterol: eGFR: estimated glomerular filtration rate. ^∗^*P* < 0.05; ^∗∗^*P* < 0.01.

**Table 3:** Main correlations of anthropometric and eGFR in the subgroup divided by sex.

Parameter	Men (*n* = 81)		Women (*n* = 90)	
	*r*	*P*	*r*	*P*
Age (year)	-0.658	<0.001	-0.546	<0.001
Diabetes duration (year)	-0.441	<0.001	-0.262	0.014
FPG (mmol/l)	0.249	0.031	0.137	0.214
UA (*μ*mol/l)	0.085	0.469	-0.305	0.005
TG (mmol/l)	0.150	0.193	-0.118	0.280
TC (mmol/l)	0.287	0.013	-0.127	0.243
HDL-c (mmol/l)	0.123	0.297	-0.042	0.705
LDL-c (mmol/l)	0.273	0.017	-0.118	0.284
HbA1c (mmol/l)	0.376	0.001	0.300	0.006
BMI (kg/m^2^)	0.164	0.157	0.139	0.201

VFA (cm^2^)	-0.045	0.758	0.079	0.580
WC (cm)	0.076	0.510	-0.075	0.492
PrFT (cm)	-0.264	0.019	-0.199	0.062
PnFT (cm)	-0.072	0.527	-0.048	0.657

eGFR: estimated glomerular filtration rate; FPG: fast plasma glucose; UA: uric acid; TG: triglyceride; TC: total cholesterol; HDL-c: high-density lipoprotein-cholesterol; LDL-c: low-density lipoprotein-cholesterol; HbA1c: glycated hemoglobin; BMI: body mass index; VFA: visceral fat area; WC: waist circumference; PrFT: perirenal fat thickness; PnFT: paranephric fat thickness.

**Table 4:** Independent multivariate correlates of eGFR in men.

Parameter	Unstandardized coefficient (B)	Standardized coefficient *β*	*P*
Model (*R*^2^ = 0.513)			
PrFT (cm)	-12.506	-0.203	0.017
Age (year)	-1.076	-0.593	<0.001
HbA1c (%)	2.674	0.186	0.034

eGFR: estimated glomerular filtration rate; PrFT: perirenal fat thickness; HbA1c: glycated hemoglobin.

## Data Availability

The data used to support the findings of this study are available from the corresponding author upon request.

## Supplementary Materials

Supplementary Materials Supplementary Table 1: characteristics of the population divided by sex.

## References

[1] Cornier M. A., Marshall J. A., Hill J. O., Maahs D. M., Eckel R. H. (2011). Prevention of overweight/obesity as a strategy to optimize cardiovascular health. *Circulation*.

[2] Dong Y., Wang Z., Chen Z. (2018). Comparison of visceral, body fat indices and anthropometric measures in relation to chronic kidney disease among Chinese adults from a large scale cross-sectional study. *BMC nephrology*.

[3] Lakkis J. I., Weir M. R. (2018). Obesity and kidney disease. *Progress in cardiovascular diseases*.

[4] Meguro S., Kabeya Y., Tanaka K. (2013). Past obesity as well as present body weight status is a risk factor for diabetic nephropathy. *International Journal of Endocrinology*.

[5] Chen H.-M., Shen W.-W., Ge Y.-C., Zhang Y.-D., Xie H.-L., Liu Z.-H. (2013). The relationship between obesity and diabetic nephropathy in China. *BMC Nephrology*.

[6] He Y., Li F., Wang F., Ma X., Zhao X., Zeng Q. (2016). The association of chronic kidney disease and waist circumference and waist-to-height ratio in Chinese urban adults. *Medicine*.

[7] Lu J. L., Kalantar-Zadeh K., Ma J. Z., Quarles L. D., Kovesdy C. P. (2014). Association of body mass index with outcomes in patients with CKD. *Journal of the American Society of Nephrology*.

[8] Duan Y., Wang X., Zhang J. (2019). Body mass index is an independent predictive factor for kidney function evaluated by glomerular filtration rate in a community-dwelling population. *Eating and weight disorders*.

[9] Nevill A. M., Stewart A. D., Olds T., Holder R. (2006). Relationship between adiposity and body size reveals limitations of BMI. *American Journal of Physical Anthropology*.

[10] Heymsfield S. B., Scherzer R., Pietrobelli A., Lewis C. E., Grunfeld C. (2009). Body mass index as a phenotypic expression of adiposity: quantitative contribution of muscularity in a population-based sample. *International Journal of Obesity*.

[11] Gómez-Ambrosi J., Silva C., Galofré J. C. (2012). Body mass index classification misses subjects with increased cardiometabolic risk factors related to elevated adiposity. *International Journal of Obesity*.

[12] Harris R. B. S., Leibel R. L. (2008). Location, location, location…. *Cell Metabolism*.

[13] Noori N., Hosseinpanah F., Nasiri A. A., Azizi F. (2009). Comparison of overall obesity and abdominal adiposity in predicting chronic kidney disease incidence among adults. *Journal of Renal Nutrition*.

[14] Kelsey R. (2013). Body fat distribution and renal risk. *Nature reviews. Nephrology*.

[15] Chen H., Liu Z., Li S. (2008). The relationship between body fat distribution and renal damage in Chinese with obesity. *Experimental and Clinical Endocrinology & Diabetes*.

[16] Pinto-Sietsma S.-J., Navis G., Janssen W. M. T., de Zeeuw D., Gans R. O. B., de Jong P. E. (2003). A central body fat distribution is related to renal function impairment, even in lean subjects. *American Journal of Kidney Diseases*.

[17] Liu B.-X., Sun W., Kong X.-Q. (2018). Perirenal fat: a unique fat pad and potential target for cardiovascular disease. *Angiology*.

[18] Cronan J. J., Yoder I. C., Amis E. S., Pfister R. C. (1982). The myth of anechoic renal sinus fat. *Radiology*.

[19] Kim J.-H., Han E.-H., Jin Z.-W. (2012). Fetal topographical anatomy of the upper abdominal lymphatics: its specific features in comparison with other abdominopelvic regions. *The Anatomical Record: Advances in Integrative Anatomy and Evolutionary Biology*.

[20] Hausman G. J. (1985). Anatomical and enzyme histochemical differentiation of adipose tissue. *International Journal of Obesity*.

[21] Czaja K., Kraeling R., Klimczuk M., Franke-Radowiecka A., Sienkiewicz W., Lakomy M. (2002). Distribution of ganglionic sympathetic neurons supplying the subcutaneous, perirenal and mesentery fat tissue depots in the pig. *Acta Neurobiologiae Experimentalis*.

[22] Jespersen N. Z., Feizi A., Andersen E. S. (2019). Heterogeneity in the perirenal region of humans suggests presence of dormant brown adipose tissue that contains brown fat precursor cells. *Molecular Metabolism*.

[23] Hartman A. D. (1985). Adipocyte fatty acid mobilization in vivo: effects of age and anatomical location. *Lipids*.

[24] Spit K. A., Muskiet M. H. A., Tonneijck L. (2020). Renal sinus fat and renal hemodynamics: a cross-sectional analysis. *Magnetic Resonance Materials in Physics, Biology and Medicine*.

[25] Notohamiprodjo M., Goepfert M., Will S. (2020). Renal and renal sinus fat volumes as quantified by magnetic resonance imaging in subjects with prediabetes, diabetes, and normal glucose tolerance. *PloS one*.

[26] Wagner R., Machann J., Guthoff M. (2017). The protective effect of human renal sinus fat on glomerular cells is reversed by the hepatokine fetuin-A. *Scientific Reports*.

[27] Krievina G., Tretjakovs P., Skuja I. (2016). Ectopic adipose tissue storage in the left and the right renal sinus is asymmetric and associated with serum kidney injury molecule-1 and fibroblast growth factor-21 levels increase. *EBioMedicine*.

[28] Foster M. C., Hwang S.-J., Porter S. A., Massaro J. M., Hoffmann U., Fox C. S. (2011). Fatty kidney, hypertension, and chronic kidney disease: the Framingham Heart Study. *Hypertension*.

[29] Grima P., Guido M., Zizza A., Chiavaroli R. (2010). Sonographically measured perirenal fat thickness: an early predictor of atherosclerosis in HIV-1-infected patients receiving highly active antiretroviral therapy?. *Journal of Clinical Ultrasound*.

[30] De Pergola G., Campobasso N., Nardecchia A. (2015). Para- and perirenal ultrasonographic fat thickness is associated with 24-hours mean diastolic blood pressure levels in overweight and obese subjects. *Bmc Cardiovascular Disorders*.

[31] Hou N., Han F., Wang M. (2014). Perirenal fat associated with microalbuminuria in obese rats. *International Urology and Nephrology*.

[32] Ma S., Zhu X.-Y., Eirin A. (2016). Perirenal fat promotes renal arterial endothelial dysfunction in obese swine through tumor necrosis factor-*α*. *The Journal of Urology*.

[33] Geraci G., Zammuto M. M., Mattina A. (2018). Para-perirenal distribution of body fat is associated with reduced glomerular filtration rate regardless of other indices of adiposity in hypertensive patients. *The Journal of Clinical Hypertension*.

[34] Sun X., Han F., Miao W., Hou N., Cao Z., Zhang G. (2013). Sonographic evaluation of para- and perirenal fat thickness is an independent predictor of early kidney damage in obese patients. *International Urology and Nephrology*.

[35] Lamacchia O., Nicastro V., Camarchio D. (2011). Para- and perirenal fat thickness is an independent predictor of chronic kidney disease, increased renal resistance index and hyperuricaemia in type-2 diabetic patients. *Nephrology Dialysis Transplantation*.

[36] Zhao Y., Gao P., Sun F. (2016). Sodium intake regulates glucose homeostasis through the PPAR*δ*/adiponectin- mediated SGLT2 pathway. *Cell Metabolism*.

[37] Schaffer J. E. (2003). Lipotoxicity: when tissues overeat. *Current Opinion in Lipidology*.

[38] Li H., Li M., Liu P. (2016). Telmisartan ameliorates nephropathy in metabolic syndrome by reducing leptin release from perirenal adipose tissue. *Hypertension*.

[39] de Vries A. P. J., Ruggenenti P., Ruan X. Z. (2014). Fatty kidney: emerging role of ectopic lipid in obesity-related renal disease. *The Lancet Diabetes & Endocrinology*.

[40] Lindström P., Wadström J., Ollerstam A., Johnsson C., Persson A. E. G. (2003). Effects of increased intra-abdominal pressure and volume expansion on renal function in the rat. *Nephrology Dialysis Transplantation*.

[41] Harman P. K., Kron I. L., Mclachlan H. D., Freedlender A. E., Nolan S. P. (1982). Elevated intra-abdominal pressure and renal function. *Annals of Surgery*.

[42] Favre G., Grangeon-Chapon C., Raffaelli C., François-Chalmin F., Iannelli A., Esnault V. (2017). Perirenal fat thickness measured with computed tomography is a reliable estimate of perirenal fat mass. *PloS one*.

[43] Ogawa H., Fujitani K., Tsujinaka T. (2011). InBody 720 as a new method of evaluating visceral obesity. *Hepato-Gastroenterology*.

