# Supplementary material for: The Relationship between Perirenal Fat Thickness and Reduced Glomerular Filtration Rate in Patients with Type 2 Diabetes
Source: J Diabetes Res. 2020 Jun 28;2020:6076145. doi: 10.1155/2020/6076145 (PMC7341433; doi:10.1155/2020/6076145)
Supplement: Supplementary Materials — Supplementary Table 1: characteristics of the population divided by sex. [file 6076145.f1.pdf]

Supplementary Table 1. Characteristics of the the population divided by sex.

	Men	Women	*P*
Age (year)	53.23 ± 17.03	62.54 ± 14.44	0.000
Diabetes duration (year)	7.00 (2.00 - 13.5)	11.50 (4.75 - 17.25)	0.006
BMI (kg/m^2)	27.25 ± 4.75	26.05 ± 4.37	0.094
WC (cm)	99.21 ± 12.37	94.70 ± 10.27	0.010
FPG (mmol/l)	8.90 ± 3.87	8.34 ± 3.74	0.349
UA (umol/l)	359.03 ± 105.97	320.00 ± 90.32	0.013
TG (mmol/l)	1.59 (1.07 - 2.69)	1.37 (1.01 - 1.78)	0.076
TC (mmol/l)	4.21 ± 1.26	4.42 ± 1.34	0.316
HDL-c (mmol/l)	0.98 ± 0.25	1.13 ± 0.28	0.000
LDL-c (mmol/l)	2.78 ± 0.95	2.86 ± 1.12	0.636
HbA1c (%)	9.25 ± 2.15	9.42 ± 2.15	0.633
VFA (cm^2)	124.29 ± 46.60	96.09 ± 30.82	0.000
PrFT (cm)	1.13 ± 0.50	0.82 ± 0.46	0.000
eGFR (ml/min/1.73 m^2)	101.01 ± 30.45	93.54 ± 31.45	0.122

BMI: body mass index, WC: waist circumference, FPG: fast plasm glucose, UA: uric acid, TG: triglyceride, TC: total cholesterol, HDL-C: high density lipoprotein-cholesterol, LDL-C: low density lipoprotein-cholesterol, HbA1c: glycated hemoglobin, VFA: visceral fat area, PrFT: perirenal fat thickness, eGFR: estimated glomerular filtration rate.
